# Impact of Social Risk Screening on Discharge Care Processes and Postdischarge Outcomes: A Pragmatic Mixed-Methods Clinical Trial During the COVID-19 Pandemic

**DOI:** 10.1097/MLR.0000000000002048

**Published:** 2024-09-06

**Authors:** Andrea S. Wallace, Alycia A. Bristol, Erin Phinney Johnson, Catherine E. Elmore, Sonja E. Raaum, Angela Presson, Kaleb Eppich, Mackenzie Elliott, Sumin Park, Benjamin S. Brooke, Sumin Park, Marianne E. Weiss

**Affiliations:** * University of Utah College of Nursing, Salt Lake City, UT; † University of Utah School of Medicine, Salt Lake City, UT; ‡ Marquette University, Milwaukee, WI

**Keywords:** social determinants of health, hospital discharge, case management, implementation evaluation, patient-reported outcomes

## Abstract

**Background:**

Social risk screening during inpatient care is required in new CMS regulations, yet its impact on inpatient care and patient outcomes is unknown.

**Objectives:**

To evaluate whether implementing a social risk screening protocol improves discharge processes, patient-reported outcomes, and 30-day service use.

**Research Design:**

Pragmatic mixed-methods clinical trial.

**Subjects:**

Overall, 4130 patient discharges (2383 preimplementation and 1747 postimplementation) from general medicine and surgical services at a 528-bed academic medical center in the Intermountain United States and 15 attending physicians.

**Measures:**

Documented family interaction, late discharge, patient-reported readiness for hospital discharge and postdischarge coping difficulties, readmission and emergency department visits within 30 days postdischarge, and coded interviews with inpatient physicians.

**Results:**

A multivariable segmented regression model indicated a 19% decrease per month in odds of family interaction following intervention implementation (OR=0.81, 95% CI=0.76–0.86, *P*<0.001), and an additional model found a 32% decrease in odds of being discharged after 2 pm (OR=0.68, 95% CI=0.53–0.87, *P*=0.003). There were no postimplementation changes in patient-reported discharge readiness, postdischarge coping difficulties, or 30-day hospital readmissions, or ED visits. Physicians expressed concerns about the appropriateness, acceptability, and feasibility of the structured social risk assessment.

**Conclusions:**

Conducted in the immediate post-COVID timeframe, reduction in family interaction, earlier discharge, and provider concerns with structured social risk assessments likely contributed to the lack of intervention impact on patient outcomes. To be effective, social risk screening will require patient/family and care team codesign its structure and processes, and allocation of resources to assist in addressing identified social risk needs.

Care transition from an acute health care facility to home is a vulnerable period for patients and an area of active quality improvement efforts given persistently low ratings among hospital quality measures.^1^ Poor care transitions also contribute to unplanned, early readmissions, which cost Medicare tens of billions annually.^2^ Health systems have adopted various modalities aimed at improving their care processes and patient outcomes to improve key drivers within value-based payment: the patient experience of discharge care, unplanned service use, and nonreimbursement for hospital readmissions. Accurate identification and anticipatory intervention with patients who have social needs that place them at risk for poor outcomes in the transition from hospital to home are crucial for effectively deploying staff and resources to prepare patients for discharge.

The hospital discharge planning, which commences upon patient admission, provides an avenue to document social risk factors in the electronic health record (EHR), making the information available to the clinical team as they prepare patients for transitions home. In fact, insights from care transition studies reveal that the degree of health team communication with patients and family members about patients’ social needs and resources at home is an important contributor to postdischarge outcomes, and discordance in understanding between nurses and patients about the home context contributes to an inability to provide adequate postdischarge planning.^3^ However, documentation in the EHR does not equate to incorporation into clinical care decisions necessary to improve postdischarge outcomes. It is well documented that the complexity and challenges of the discharge process that have only increased since the COVID-19 pandemic—declining lengths-of-stay, limited outpatient care knowledge among hospitalists, and steep nurse staffing shortages—affect the quality of discharge planning.^4–6^

Screening for social risks—inadequate food, housing, social support—is no longer merely a consensus recommendation, but rather a quality metric required by accreditation bodies and payors. As part of its Fiscal Year 2023 Hospital Inpatient Prospective Payment System (IPPS), the Centers for Medicare and Medicaid Services (CMS) is now including “Screening for Social Drivers of Health” which measures the proportion of patients who were screened for social risk factors at the time of hospital admission; and “Screen Positive Rate for Social Driver of Health” which measures the proportion of patients who were identified to have one or more social risk factors.^7^ Yet, careful studies to address the impact of social risk screening on patient outcomes remain to be realized. Here, we sought to implement an efficient, structured, low-cost intervention for assessing and communicating patients’ social risks during hospital discharge planning, testing for its effectiveness in improving discharge care processes, patient-reported outcomes on the day of discharge and postdischarge, and return to hospital for readmission or emergency department (ED) visits. We also evaluated the feasibility, acceptability, and appropriateness of the implementation of social risk screening among inpatient physicians.

## METHODS

This pragmatic integrative mixed-methods trial used a longitudinal design incorporating a 12-month preimplementation baseline data collection period, a 1-month washout-pilot period to integrate the intervention into unit workflow, and a 17-month intervention implementation period during which social risk assessment and EHR communication were conducted. Intervention implementation was staggered, starting first in surgical units and then expanding to medicine units. We compared processes of care [family interaction and late discharge (after 2 pm)] and outcomes (readiness for hospital discharge, postdischarge coping difficulty, and return to the hospital within 30 d) for patients discharged during the preimplementation period with patients discharged after the social risk screening intervention was implemented. We also conducted an evaluation of the implementation of the social risk screening intervention with attending physicians who had received social risk information during the postimplementation period. All study procedures were approved by the University of Utah Institutional Review Board (IRB #00126445).

### Setting and Sample

The study was conducted in 5 inpatient units at a 528-bed Academic Health Sciences University Hospital in the intermountain United States, examining patient discharges from 3 general medicine and 2 surgical services. Throughout the preimplementation (2/2020–1/2021) and postimplementation (2/2021–7/2022) phases, consecutive patients on participating units with a discharge order documented in the EHR during weekday hours (8 am–5 pm) were contacted through hospital room telephone. Upon having a discharge order placed in the EHR, patients in both preimplementation and postimplementation groups were contacted by study staff. Those who verbally consented were asked to complete a verbal survey regarding their perceived discharge readiness before the time of discharge. Patients were additionally asked if they were willing to electronically complete a survey 2 weeks after discharge to assess coping difficulties they may have experienced after being discharged (detailed information about the instruments is described in “Outcomes and Measurements”). Patients admitted with a primary or secondary psychiatric diagnosis or who were cognitively impaired or unable to communicate verbally were excluded; those admitted from skilled nursing facilities, or cared for by palliative/hospice, transplant, bariatric services, or for end-stage renal disease were also excluded because of the extensive social risk evaluation offered as part of those services.

Attending physicians who received social risk screening information as outlined in the study protocol were invited to participate in telephone interviews to assess implementation of the intervention.

### Intervention

The intervention protocol included 3 components: (1) systematic screening of social risks; (2) interpretation of patients’ social risks by inpatient registered nurse (RN) case managers; and (3) EHR communication of under-resourced patients to the attending physicians when patients were identified as lacking adequate homegoing resources by RN case managers.

Patient social risks were identified during the patient admission process as part of RN case manager intake assessments, using a 14-item questionnaire composed of 10 social needs (eg, food, housing, transportation) questions from the SINCERE social needs screener, and 4 questions inquiring about the availability of someone to help with transportation, errands, medication management, meals, and insurance/health forms (representing instrumental social support). The 10 SINCERE social needs questions and 4 additional instrumental support questions have been validated in preliminary studies.^8–11^ A final interpretive, resource adequacy question, “Given what you know about this individual, do you anticipate this person will have adequate personal resources at the time of discharge?” was answered by RN case managers assigned to help coordinate patient discharge.

Responses to the social risk assessment were added to an EHR discharge planning tab. An answer of “Not Likely” or “No” to the resource adequacy question answered by the RN case managers resulted in an alert banner appearing on the patient’s discharge planning tab. Research staff checked reports at 10 am every weekday for any responses of “Not Likely” or “No” and contacted patients’ attending physicians through the EHR messaging to inform them about the patient’s status and the need to check the discharge planning tab or to speak with the RN case manager for more information.

### Outcomes and Measurements

Measures of changes in discharge processes included documentation of family interaction and late discharge, which were collected from both preimplementation and postimplementation groups. Documentation of family interaction was defined as any patient for whom the “Family Update” field was marked at least once in the EHR by a member of the clinical team (ie, RN case manager or clinical RN). Late discharge was defined as discharge after 2 pm, often perceived as indicative of inefficiencies in the discharge process. We hypothesized that early assessment and discussion of patients’ social risks and available resources would enhance the efficiency of the discharge process, resulting in fewer instances of late hospital discharge. Outcome measures included patient-reported readiness for hospital discharge measured on the day of hospital discharge, patient-reported postdischarge coping difficulties in the first 2 weeks postdischarge, and return to hospital (30-day readmission and ED visit).

Patient-reported discharge readiness was assessed using the 8-item Readiness for Hospital Discharge (RHDS)^12–15^ assessing 4 dimensions of readiness that independently and together serve as outcomes of discharge preparation—personal status, knowledge, coping, and expected support. The RHDS is correlated with quality of discharge teaching, difficulties coping postdischarge, care coordination, and utilization of postdischarge services; lower perception of readiness at discharge is associated with increased use of both informal and formal support postdischarge, and readmissions.^3,6,12,16,17^ The validity of the RHDS instrument is supported by confirmatory factor analysis, contrasted group comparisons, and predictive validity testing. The RHDS uses a 0–10-point rating scale for each item with total score reported as the mean of the items (range of 0–10); higher scores indicate greater readiness.^13,14^

Patient coping difficulties was assessed with the 10-item Post-Discharge Coping Difficulty Scale (PDCDS), sent electronically through REDCap 2 weeks after discharge^16,18^ that assesses patient stress, recovery, self-care, self-medical management, family difficulty, help required, emotional support, confidence in self-care, medical management abilities, and adjustment; higher scores indicate greater coping difficulty after hospital discharge (total score=item mean ranging from 0 to 10). The PDCDS is responsive to discharge interventions.^18,19^

Return to index hospital within 30 days includes readmission coded as 0=no readmission and 1=at least 1 readmission and ED visits coded as 0=no ED visits and 1=at least 1 ED visit that did not result in a concurrent readmission.

Covariates included patient age, binary gender, race and ethnicity, insurance status, discharging hospital unit, whether the admission was planned, patient discharge destination (home, home with home health, skilled nursing facility, long-term acute care, and other), and whether the patient tested positive for COVID-19. LACE, a composite score indicating readmission risk comprised of total length of hospital stay, acuity on admission, preexisting comorbidities, and number of ED visits in the past 6 months, was included.^20^

Patient demographics and patient-reported outcomes were collected during the patient survey. Documentation of family interaction, discharges after 2 pm, admission details and clinical status were collected from the EHR. 30-day hospitalizations and ED visits were collected from the University of X Enterprise Data Warehouse (EDW). All data were managed using REDCap.^21–23^

Physician perceptions of the feasibility, acceptability, and appropriateness of the social risk screener for assessing and communicating patients’ needs were assessed using a semistructured interview (Supplemental Digital Content 1, http://links.lww.com/MLR/C871). All interviews were recorded and transcribed verbatim.

### Statistical Analysis

Patient demographics and outcome measures were summarized by preimplementation and postimplementation groups. For continuous variables, we reported mean and SD or median and interquartile range (IQR). For categorical variables, counts, and percentages were reported.

We conducted an intent-to-treat analysis, comparing preimplementation and postimplementation phases on discharge process measures (family interaction documentation, late discharges), patient-reported outcomes (readiness for hospital discharge, postdischarge coping difficulties), and postdischarge utilization (30-day readmission, ED use). Multivariable segmented regression analyses were performed to model the interrupted time series (ITS) data using generalized estimating equations (GEE) with an autoregressive (lag 1) correlation structure to account for subjects with multiple visits. The segmented regression model provides an immediate postintervention level change (indicated as “Intervention group: Post” in our results), and the difference in the intervention effect over time (“Time After”) relative to the effect over time that we would expect in the absence of an intervention(“Time”).^24^ We have chosen ITS design because it is considered the strongest among quasiexperimental designs and is a powerful tool used for evaluating the impact of interventions and programs implemented in health care settings. With this design, outcomes are measured at different time points before and after implementing an intervention, allowing the change in level and trend of outcomes to be compared, to evaluate intervention effects.^24^

Binary outcomes were analyzed using logistic regression and coefficients were exponentiated to report odds ratios (ORs) with corresponding 95% CIs and *P*-values. The RHDS outcome was left-skewed, and as a result we transformed it by subtracting observations from the maximum value in order to fit a gamma regression model; due to this transformation, higher scores indicate lower readiness. We used an identity link to provide interpretations as mean changes (Beta) to be consistent with PDCDS, which we analyzed using a normal model. Statistical significance was assessed at the 0.05 level and analyses were performed using R version 4.2.1 (https://cran.r-project.org/).

### Qualitative Analysis

Physician interviews were analyzed using content analysis.^25,26^ The coding process involved 2 phases: (1) deductive coding using an initial codebook based on concepts in the interview guide. New codes emerged during this phase and were added to the codebook after discussion by team members. Transcripts were coded by XX, XX, XX, and XX (authors, blinded); (2) authors reviewed the application of codes across the interviews to check for consistency and accuracy in the coding process. Then, codes were grouped into categories. Throughout, the team members met regularly to review the coding and category development, resolving disagreements until consensus and themes were established. Rigor in the qualitative analysis was guided by Lincoln and Guba’s trustworthiness criteria.^26^

## RESULTS

Over 28 months we enrolled N=4130 patients who completed the RHDS: 2383 (58%) in the preimplementation phase and 1747 (42%) in the postimplementation phase. Postimplementation, one-quarter (26%, n=457) of the participants underwent systematic screening of social risks conducted by the case manager. See Table 1 for participant demographics and other covariates by phase.

**TABLE 1:** Participant Demographics and Study Variables

	Overall, n (%)	Intervention group, n (%)			
Variable	N=4130	Pre, N=2383	Post, N=1747	N Missing	*P* *
Age, median (IQR)	52 (38–64)	53 (39–63)	52 (37–64)	185	0.8
Gender				186	0.047
Female	1945 (49)	1097 (48)	848 (51)		
Male	1999 (51)	1190 (52)	809 (49)		
Race				44	0.036
American Indian or AK Native	119 (2.9)	79 (3.3)	40 (2.3)		
Asian	53 (1.3)	27 (1.1)	26 (1.5)		
Black or African-American	80 (2.0)	50 (2.1)	30 (1.7)		
Other	240 (5.9)	139 (5.9)	101 (5.8)		
Other Pacific Islander	48 (1.2)	34 (1.4)	14 (0.8)		
Unknown	192 (4.7)	96 (4.1)	96 (5.6)		
White or Caucasian	3354 (82)	1934 (82)	1420 (82)		
Ethnicity				44	0.11
Hispanic/Latino	291 (7.1)	172 (7.3)	119 (6.9)		
Not Hispanic/Latino	3561 (87)	2067 (88)	1494 (87)		
Unknown	234 (5.7)	120 (5.1)	114 (6.6)		
Insurance				171	0.068
Commercial	1818 (46)	1032 (45)	786 (47)		
Medicaid	743 (19)	453 (20)	290 (17)		
Medicare	1216 (31)	697 (31)	519 (31)		
Other	103 (2.6)	58 (2.5)	45 (2.7)		
Workers comp	79 (2.0)	36 (1.6)	43 (2.6)		
Planned admission	1294 (32)	638 (27)	656 (38)	24	<0.001
COVID positive	387 (9.8)	262 (12)	125(7.2)	175	<0.001
LACE total score, median (IQR)	7.0 (5.0–10.0)	8.0 (5.0–11.0)	7.0 (5.0–10.0)	45	<0.001
Hospital unit				0	<0.001
Surgical A	940 (23)	375 (16)	565 (32)		
Surgical B	1456 (35)	687 (29)	769 (44)		
Medicine A	551 (13)	422 (18)	129 (7.4)		
Medicine B	727 (18)	579 (24)	148 (8.5)		
Medicine C	456 (11)	320 (13)	136 (7.8)		
Discharge destination				19	0.2
Home	2953 (72)	1685 (71)	1268 (73)		
Home health	767 (19)	443 (19)	324 (19)		
Skilled nursing	308 (7.5)	187 (7.9)	121 (7.0)		
Long-term acute care	19 (0.5)	13 (0.5)	6 (0.3)		
Other	64 (1.6)	44 (1.9)	20 (1.2)		
Family interaction documented	1978 (69)	1023 (67)	955 (72)	1264	0.004
Discharged after 2 pm	2408 (59)	1403 (59)	1005 (58)	45	0.4
RHDS total, median, n (IQR)	8.75 (7.50–9.38)	8.75 (7.63–9.38)	8.63 (7.50–9.38)	0	0.2
PDCDS total, median, n (IQR)	2.90 (1.50–4.30)	2.70 (1.35–4.30)	3.10 (1.80–4.40)	3377	0.063
30-day rehospitalization	926 (23)	629 (27)	297 (17)	44	<0.001
30-day ED visit	565 (14)	370 (16)	195 (11)	44	<0.001

^*^ Wilcoxon rank sum test; Pearson’s χ^2^ test.

ED indicates emergency department; IQR, interquartile range; LACE, LACE Index Scoring Tool for Risk Assessment of Death and Readmission; PDCDS, Post-Discharge Coping Difficulties Scale; RHDS, Readiness for Hospital Discharge Scale.

Fifty-one physicians were invited for implementation evaluation interviews; 15 (29%) agreed to participate. For physician participant demographics see Supplemental Digital Content 2, http://links.lww.com/MLR/C872.

### Discharge Processes Preimplementation and Postimplementation of Social Risk Screening

The postimplementation phase was characterized by a 19% decrease per month in odds of family interaction following intervention implementation (OR=0.81, 95% CI=0.76–0.86, *P*<0.001). The postimplementation period was associated with lower likelihood of documented family interaction with increasing age, among Black or African American patients, and those with a planned admission. Two units (Surgical Unit B, Medicine Unit A) experienced lower postimplementation documentation of family interaction than the reference category unit (Surgical Unit A). Conversely, those with higher LACE scores and those with COVID-19 were more likely to have family interaction documented.

In the postimplementation phase, there was an immediate 32% decrease in odds of late discharge (OR=0.68, 95% CI=0.53–0.87, *P*=0.002). Late discharges were less likely for those with planned admissions and discharges from 2 of the medicine units. Conversely, those with higher LACE scores, and discharged from Surgical Unit B, to home health, skilled nursing, and long-term acute care were more likely to be discharged after 2 pm (Table 2).

**TABLE 2:** Segmented Regression of Process Measures: Documented Family Interaction and Discharge After 2 pm

	Documented Family Interaction (N=2496)		Discharge After 2 pm (N=3619)	
Characteristic	OR (95% CI)	*P*	OR (95% CI)	*P*
Intervention group				
Pre	—		—	
Post	1.28 (0.82–2.01)	0.3	0.68 (0.53–0.87)	0.003
Time*	1.18 (1.12–1.24)	<0.001	1.02 (1.00–1.05)	0.091
Time after*	0.81 (0.76–0.86)	<0.001	1.01 (0.98–1.05)	0.5
Age	0.99 (0.98–1.00)	0.010	1.00 (0.99–1.00)	0.2
Gender				
Female	—		—	
Male	0.83 (0.67–1.04)	0.11	0.94 (0.82–1.08)	0.4
Ethnicity and race				
White non-Hispanic	—		—	
White Hispanic	0.78 (0.40–1.50)	0.5	1.17 (0.73–1.87)	0.5
American Indian or AK Native	1.10 (0.50–2.45)	0.8	1.21 (0.79–1.85)	0.4
Black or African-American	0.37 (0.17–0.83)	0.015	1.15 (0.72–1.84)	0.6
Pacific Islander	1.61 (0.32–8.17)	0.6	1.14 (0.59–2.22)	0.7
Other/unknown	1.19 (0.79–1.81)	0.4	1.00 (0.78–1.27)	>0.9
Insurance				
Commercial	—		—	
Medicaid	0.72 (0.52–1.01)	0.056	1.15 (0.95–1.40)	0.2
Medicare	0.89 (0.65–1.21)	0.5	1.00 (0.83–1.21)	>0.9
Other	0.74 (0.38–1.45)	0.4	1.24 (0.80–1.90)	0.3
Workers comp	1.38 (0.72–2.65)	0.3	1.02 (0.61–1.70)	>0.9
Planned admission	0.56 (0.43–0.73)	<0.001	0.63 (0.52–0.76)	<0.001
COVID positive	2.21 (1.05–4.65)	0.037	1.32 (0.95–1.83)	0.10
LACE total acuity score	1.10 (1.05–1.14)	<0.001	1.03 (1.00–1.05)	0.034
Hospital unit				
Surgical A	—		—	
Surgical B	0.08 (0.06–0.11)	<0.001	1.44 (1.19–1.73)	<0.001
Medicine A	0.16 (0.09–0.30)	<0.001	0.89 (0.64–1.25)	0.5
Medicine B	1.18 (0.66–2.10)	0.6	0.71 (0.54–0.93)	0.013
Medicine C	0.50 (0.25–1.02)	0.057	0.73 (0.54–0.98)	0.039
Discharge destination				
Home	—		—	
Home health	1.10 (0.82–1.48)	0.5	1.78 (1.48–2.15)	<0.001
Skilled nursing	0.88 (0.56–1.37)	0.6	1.57 (1.18–2.09)	0.002
Long-term acute care	2.53 (0.67–9.60)	0.2	6.38 (1.42–28.6)	0.016
Other	2.18 (0.96–4.95)	0.063	1.27 (0.67–2.41)	0.5

^*^ The trend in practice over time (ie, slope) before intervention; time after, the difference in slope between the preimplementation and postimplementation slopes.

LACE indicates LACE Index Scoring Tool for Risk Assessment of Death and Readmission; OR, odds ratio.

### Patient-Reported Discharge Readiness and Postdischarge Coping Difficulties Preimplementation and Postimplementation of Social Risk Screening

Overall, there were no postimplementation changes in patient-reported discharge readiness (β=−0.11, 95% CI=−0.27, 0.05, *P*=0.2). There were, however, differences for several covariates. Lower discharge readiness was associated with being African American, having Medicaid insurance versus Commercial insurance, higher LACE acuity score, and those discharged to supportive care versus home (Table 3). Conversely, higher discharge readiness was associated with increasing age, male gender, being Pacific Islander versus White non-Hispanic, having a planned admission, and being COVID positive (Table 3). Likewise, there were no postimplementation changes in postdischarge coping difficulties (β=0.14, 95% CI=−0.40 to 0.68, *P*=0.6). Increasing age, male gender, Pacific Islander race, Medicare insurance, higher LACE acuity score, and discharge to long-term acute care report fewer coping difficulties. Discharge to skilled nursing facility was associated with fewer coping difficulties. We observed variation in the associations of hospital units with both RHDS and PDCDS (Table 3).

**TABLE 3:** Segmented Regression of Patient Readiness for Hospital Discharge Scale (RHDS) Post-Discharge Coping Difficulties Scale (PDCDS)

	RHDS multivariable^*^ (N=3680)			PDCDS multivariable^†^ (N=681)		
Characteristic	Beta	95% CI	*P*	Beta	95% CI	*P*
Group						
Pre	—	—		—	—	
Post	−0.11	−0.27 to 0.05	0.2	0.14	−0.40 to 0.68	0.6
Time	0.00	−0.02 to 0.01	0.9	−0.01	−0.06 to 0.05	0.8
Time after	0.02	0.00–0.05	0.053	0.07	−0.01 to 0.15	0.10
Age	−0.01	−0.01, 0.00	<0.001	−0.03	−0.04 to−0.02	<0.001
Gender						
Female	—	—		—	—	
Male	−0.10	−0.20 to −0.01	0.038	−0.54	−0.82 to −0.27	<0.001
Ethnicity and race						
White non-Hispanic	—	—		—	—	
White Hispanic	−0.19	−0.50 to 0.13	0.2	0.54	−0.72 to 1.8	0.4
American Indian and AK Native	0.17	−0.12 to 0.46	0.2	0.02	−1.0 to 1.1	>0.9
Black or African-American	0.71	0.29 to 1.1	<0.001	−0.31	−1.5 to 0.84	0.6
Pacific Islander	−0.66	−1.0 to −0.31	<0.001	−1.2	−2.1 to −0.34	0.007
Other/unknown	−0.09	−0.26 to 0.07	0.3	−0.45	−0.96 to 0.06	0.081
Insurance						
Commercial	—	—		—	—	
Medicaid	0.48	0.32–0.63	<0.001	0.51	−0.03 to 1.1	0.063
Medicare	0.18	0.06–0.29	0.002	0.38	0.01–0.75	0.047
Other	0.12	−0.14 to 0.38	0.4	0.63	−0.18 to 1.4	0.13
Workers comp	0.16	−0.21 to 0.53	0.4	0.97	0.20–1.7	0.014
Planned admission	−0.11	−0.21 to −0.01	0.034	−0.09	−0.46 to 0.28	0.6
COVID positive	−0.30	−0.45 to −0.16	<0.001	−0.63	−1.3 to 0.05	0.069
LACE total acuity score	0.03	0.02–0.05	<0.001	0.07	0.02–0.13	0.008
Hospital unit						
Surgical A	—	—		—	—	
Surgical B	0.12	−0.02 to 0.25	0.087	0.84	0.53–1.2	<0.001
Medicine A	−0.16	−0.32 to 0.00	0.047	0.94	0.19–1.7	0.014
Medicine B	0.09	−0.07 to 0.25	0.3	0.93	0.36–1.5	0.001
Medicine C	−0.11	−0.29 to 0.06	0.2	0.31	−0.32 to 0.95	0.3
Discharge destination						
Home	—	—		—	—	
Home health	0.19	0.07–0.31	0.002	1.0	0.60–1.4	<0.001
Skilled nursing	1.4	1.2–1.7	<0.001	1.5	0.33–2.6	0.011
Long-term acute care	1.1	0.12–2.1	0.028	−2.6	−3.2 to −2.0	<0.001
Other	1.3	0.83–1.9	<0.001	2.3	−1.1 to 5.8	0.2

^*^ RHHDS scores are transformed; higher score indicated lower readiness.

^†^ PDCDS scores; higher scores indicate lower postdischarge coping difficulty.

LACE indicates LACE Index Scoring Tool for Risk Assessment of Death and Readmission; time, the trend in practice over time (ie, slope) before intervention; time after, the difference in slope between the preimplementation and postimplementation slopes.

### Thirty-Day Hospital Readmissions and ED Visits Preimplementation and Postimplementation of Social Risk Screening

While the 30-day readmission rate were 27% (n=640) preimplementation and 17% (n=297) postimplementation (Table 1), social risk screening was not associated with reductions in hospital readmissions (OR: 0.91, 95% CI=0.56–1.46, *P*=0.7; Table 4). However, among the covariates, male gender, LACE total score, and discharge to home health and other settings (vs. home or supportive care) were associated with higher odds of readmission.

**TABLE 4:** Multivariable Segmented Regression for 30-Day Rehospitalization and Emergency Department Readmission

	Hospitalization (N=3626)		ED (N=3626)	
Characteristic	OR (95% CI)	*P*	OR (95% CI)	*P*
Intervention group				
Pre	—		—	
Post	0.91 (0.56–1.46)	0.7	0.74 (0.44–1.25)	0.3
Time	0.99 (0.95–1.03)	0.5	1.04 (0.99–1.09)	0.2
Time after	1.03 (0.96–1.11)	0.3	0.97 (0.90–1.05)	0.5
Age	0.99 (0.98–1.00)	0.059	0.99 (0.98–1.00)	0.018
Gender				
Female	—		—	
Male	1.36 (1.04–1.78)	0.023	0.92 (0.69–1.23)	0.6
Ethnicity and race				
White non-Hispanic	—		—	
White Hispanic	0.90 (0.37–2.16)	0.8	0.79 (0.30–2.10)	0.6
American Indian and AK Native	1.18 (0.60–2.30)	0.6	1.00 (0.51–1.95)	>0.9
Black or African-American	1.03 (0.43–2.46)	>0.9	0.68 (0.24–1.96)	0.5
Pacific Islander	0.60 (0.18–1.99)	0.4	0.81 (0.23–2.93)	0.8
Other/unknown	0.81 (0.50–1.33)	0.4	1.49 (0.97–2.28)	0.069
Insurance				
Commercial	—		—	
Medicaid	0.77 (0.53–1.12)	0.2	2.36 (1.65–3.37)	<0.001
Medicare	0.82 (0.56–1.20)	0.3	1.95 (1.31–2.92)	0.001
Other	0.95 (0.37–2.42)	>0.9	1.14 (0.37–3.51)	0.8
Workers comp	1.59 (0.76–3.33)	0.2	0.52 (0.07–4.05)	0.5
Planned admission	0.78 (0.51–1.21)	0.3	0.70 (0.42–1.17)	0.2
COVID positive	0.89 (0.53–1.51)	0.7	0.84 (0.49–1.47)	0.6
LACE total	1.23 (1.18–1.28)	<0.001	1.24 (1.19–1.31)	<0.001
Hospital unit				
Surgical A	—		—	
Surgical B	0.96 (0.65–1.43)	0.9	0.84 (0.54–1.31)	0.4
Medicine A	0.81 (0.46–1.43)	0.5	0.82 (0.45–1.50)	0.5
Medicine B	1.20 (0.74–1.97)	0.5	0.65 (0.38–1.13)	0.13
Medicine C	0.66 (0.37–1.18)	0.2	0.43 (0.23–0.80)	0.008
Discharge destination				
Home	—		—	
Home health	1.66 (1.21–2.28)	0.002	1.38 (0.96–1.98)	0.081
Skilled nursing	1.10 (0.65–1.87)	0.7	0.71 (0.40–1.28)	0.3
Long-term acute care	1.14 (0.22–5.85)	0.9	0.98 (0.18–5.27)	>0.9
Other	8.97 (4.59–17.5)	<0.001	0.62 (0.18–2.11)	0.4
RHDS	1.00 (0.99–1.02)	0.5	1.00 (0.98–1.01)	0.5

ED indicates emergency department; LACE, LACE Index Scoring Tool for Risk Assessment of Death and Readmission; OR, odds ratio; RHDS, Readiness for Hospital Discharge Scale; time, the trend in practice over time (ie, slope) before intervention; time after, the difference in slope between the preimplementation and postimplementation slopes.

There were no significant changes in the number of those with one or more ED visits within 30 days postdischarge (OR: 0.74, 95% CI: 0.44–1.25, *P*=0.3; Table 4). Those with Medicaid and Medicare and those with a higher LACE total score were more likely to have visited the ED within 30 days of discharge (Table 4).

### Physician Implementation Experiences

Participating physicians identified 3 themes regarding how they viewed the acceptability, appropriateness, and feasibility of social risk screening during inpatient care. These themes included (1) feeling responsible while relying on a team; (2) preference for informal processes when communicating social risks; and (3) desire for autonomy and lack of self-efficacy when addressing social risks. See Table 5 for exemplar quotes.

**TABLE 5:** Inpatient Physician Experiences With and Opinions of Social Risk Screening

Qualitative themes	Exemplar quotes
Theme 1: Feeling responsible while relying on a team	“As an attending surgeon, I need to make sure that care continues after the hospital.” (#003) “Whenever I’m discharging someone, I have a responsibility to ensure that I’m discharging them to a safe place. Part of that is an assessment of their physical and social needs and, the context of the environment from which they came into the hospital.” (#006) “I think that that’s often something that requires additional staff support to fully get all of that necessary information” (#006) “I rely upon case managers and social workers to interface with resources my patients are going to need at home.” (#001)
Theme 2: Preference for informal processes when communicating social risks	“Sometimes it’s from the case management notes. And usually, then, it leads to more in-depth discussions with patients. On a rare occasion, it may come from talking to the patient first.” (001) “We have our APCs [Advanced Practice Clinicians] meet with discharge planning and others three times a week before rounds so that we have the information that’s relevant in terms of discharge planning. And so, that’s how that information is conveyed.” (004) “There is various information in the chart about patients and their social needs ... I look at the case manager’s notes. I look at the therapist’s notes” (012). “Whenever I have a sense that somebody’s from a different state or has a lot of post-discharge needs, then I make it a point to ask a patient, and then also just bring it back to the case management to make sure that they’re aware of that.” (005)
Theme 3: Desire for autonomy and lack of self-efficacy when addressing social risks	“we don’t use a standard screening tool *per se* on every single patient ... ⁠— nor do I necessarily think we should, because it gets really confusing and complicated. And then before you know it, you’re like, ‘well, we’re using this screening tool to use this other screening tool.’ Then you’ve got like seven different screening tools and like look ⁠— I kind of just need to ask about why they got here in the hospital…I mean, I’m looking at this is a lot of questions. And so, I imagine if there’s, like, you know, I don’t know a well-dressed family member coming in with double private insurance and … I probably don’t ⁠—probably don’t need to ask this person, right?” (010) “I think social information is a really broad topic…I think lumping everything together from like, family needs to smoking cessation and substance abuse is really like those are different things.” (005) “I feel very limited in what my actual abilities are… I can kind of coordinate care with the social worker, with the case manager, you know, sometimes the patients’ families. However, you know, I feel kind of vastly limited in what I can actually do to support people who don’t have housing, appropriate insurance, or food.” (009)

Theme 1: Feeling responsible while relying on a team related to acceptability of the intervention, defined as whether the intervention is viewed as agreeable, palatable, or satisfactory.^27^ Physicians felt responsible for understanding both patients’ medical status and their potential needs at home, and discussed how their decision-making process during discharge planning centered around their ability to understand how patients would fare postdischarge. However, physicians also identified confusion regarding who should specifically conduct social risk assessments and identified reliance upon other members of the health care team to gather information and support their ability to guide patients and families through discharge.

Theme 2: Preference for informal processes when communicating social risks related to appropriateness of the intervention, or how well the intervention fits the practice setting and provider to address a particular problem.^27^ When physicians were asked about their experiences communicating social risk information, they denied extensive engagement with the EHR system. Instead, they shared multiple examples of processes that existed before the intervention protocol that they found more conducive to workload. Overall, physicians identified that an informal process of communication occurred in the form of discussion during individual and team discharge meetings.

Theme 3: Desire for autonomy and lack of self-efficacy when addressing social risks related to feasibility of the intervention, or the extent to which intervention can be successfully used or carried out within a given setting.^27^ While physicians highlighted existing informal communication processes, most identified barriers they encountered when considering patients’ social risk information. Physicians acknowledged that patients with social risks required more intensive consideration to ensure safe and successful transitions. However, they expressed concerns about how structured social risk screening may compromise their autonomy, how the breadth of important social information may not be identified by one single, standardized assessment, and being torn about asking questions when they knew they would be unable to address identified social needs.

## DISCUSSION

This study implemented a systematic process for assessing patients’ social risks, interpreting home resource adequacy by RN case managers, and communicating findings through the EHR to inpatient attending physicians involved in hospital discharge planning. While we found no effects on patient-reported outcomes (patient readiness for discharge, postdischarge coping), or on 30-day rehospitalizations or ED visits, there was evidence of both beneficial and concerning changes in discharge processes, specifically a reduction in late discharges and a decrease in documented family interactions with the health care team. While the use of the social risk screening process could indicate that it may help optimize patients’ discharge preparation through anticipatory intervention by the clinical team, an alternate interpretation is that expedited discharge and limited family interaction were consequences that resulted from, and lingered beyond, restrictions imposed during COVID-19. Despite careful attention to assure communication of social risks to attending physicians through the EHR and with the case manager and study team, we found little evidence of physician engagement with the structured social risk assessment for communication of social risk and needs information and physician preferences for the established informal discharge communication format.

While the intervention was not as intensive as many aiming to improve discharge quality, the lack of significant impact of social risk assessment on patient-reported outcomes and service use is likely explained by limited penetration of the intervention due to COVID-related disruptions: contact restrictions required that the intervention rely on the EHR, and implementation was done during a time of extreme system disruptions and stress on staff. In other words, we aimed to improve assessment and communication of patients’ social risks during a time characterized by generalized breakdowns in team communication, poorer patient-team communication, and rush to discharge. In fact, while all intervention activities took place during the COVID-19 pandemic, reduction in family interaction documentation before and after the social risk assessments were implemented likely provides additional evidence of how COVID-related pressures and contact restrictions affected patient-team communication.

Our social risk assessment was conducted with RN case managers whose focus is on identifying postdischarge needs and engaging in anticipatory planning with families and interdisciplinary teams. While this is a subject beyond the analyses presented above, it would be reasonable to believe that, even if no benefit to readmissions or ED use, the intervention may have improved the identification of risks in patients’ home environments. However, even within this well-resourced environment, we found no evidence that communication about social risks increased with a structured screening approach, and there was no change in patient-reported outcomes that would indicate improved communication between discharge teams, patients, and families. Rather, our results demonstrated that readmissions were largely driven by patient acuity, as represented by the LACE score, and that insurance and, to a lesser degree, race/ethnicity were associated with the outcomes explored throughout this study. It is important to note that there is the possibility these covariates may—either knowingly or unknowingly—serve as a proxy for social risk by the RN case managers, an idea that is supported by physicians’ desire to tailor assessments based on what they view as risk factors. Further examination of factors associated with RN case manager interpretation of home resource adequacy, and whether and how it is associated with discharge decision-making and patient outcomes, is warranted. This may be particularly important given case managers’ role in procuring postdischarge services and the fact that our data demonstrate lower discharge readiness among patients discharged to settings other than home. Moreover, while evaluating social risks, assessing available resources is essential elements of the Transitional Care Model for improving patient outcomes, a thorough exploration of additional components is warranted.^28^ This includes fostering a trustworthy relationship between health care providers and patients, actively involving patients and caregivers in the care plan, and ensuring seamless communication coordination between hospital and community-based staff members. Furthermore, it is essential to promote continuity of care coordination from hospital to home to prevent interruptions in care across various settings and enhance patient outcomes.^28^

Considering the intervention’s focus on social risk communication, the lack of impact on service use is not altogether surprising. While meta-analyses and systematic reviews have reported that a number of interventions are effective at reducing hospital readmissions,^29–32^ even for multimodal, propensity-matched control, and RCTs,^33,34^ a reduction in readmissions and ED visits has been elusive. As offered in a landmark National Academies 2019 publication, an approach for addressing social determinants is to couple screening for patients’ specific social needs—for housing, food, transportation—with referrals to appropriate resources and support services,^35^ and is why the second question in the CMS regulations focuses on connecting to resources.^7^ We propose that social risk screening is an important tool whose full utility will only be realized as part of a coordinated systematic care delivery system able to implement direct patient-centered interventions to alleviate social needs identified by the clinical team. While the question of whether or not the prescription and execution of the social risk modifiers should be managed by the inpatient clinical team is beyond the scope of this study, findings from this study’s physician interviews suggest that, without a comprehensive approach for managing care transitions, the intensive, and challenging requirement to document social risk screening may not be balanced by its benefit.

## LIMITATIONS

The significant disruptions due to the COVID-19 pandemic on inpatient care, including the key process of health care team communication, and this study cannot be understated. While our overall readmission rate was adequate to explore the intervention’s effect, our sample limited the number of covariates that could be explored, particularly related to the caregiver interaction and postdischarge coping models where the decreased sample size from nonresponse which could bias the results. And, finally and perhaps most importantly, we have evidence that the intervention had limited penetration with 26% of those targeted having social risks acknowledged by an RN case manager in the EHR and no tracking of risk-mitigating actions taken by the health care team. These findings, along with our qualitative findings, suggests there may be bias in who was screened (ie, patients screened may have a greater likelihood of readmission than general population), and will be a key consideration in future studies exploring the nature and effect of social risk screening in inpatient settings.

## CONCLUSION

Integrating social risk screening and team communication into the patient EHR was associated with limited process changes, and no patient-reported or utilization outcome improvements. We found little qualitative evidence of impact on team communication. Our findings strongly suggest that there will be little effect of structured social risk screening on inpatient processes and outcomes without interventions coupling social risk screening with both clinician-directed and patient-directed resources both before and after hospital discharge.

## Supplementary Material

## References

[1] Centers for Medicare & Medicaid Services Health Services Advisor Group. Summary of HCAHPS Survey Results July 2021 to June 2022 Discharges. Accessed July 25, 2023. https://www.hcahpsonline.org

[2] Agency for Healthcare Research and Quality. Overview of Clinical Conditions With Frequent and Costly Hospital Readmissions by Payer, 2018. Vol. 278, 2021. HCUP Statistical Brief. www.hcup-us.ahrq.gov/reports/statbriefs/sb278-Conditions-Frequent-Readmissions-By-Payer-2018.pdf 34460186

[3] Opper K Beiler J Yakusheva O. Effects of implementing a health team communication redesign on hospital readmissions within 30 days. Worldviews Evid Based Nurs. 2019;16:121–130. 30919571 10.1111/wvn.12350

[4] Harrison JD Greysen RS Jacolbia R. Not ready, not set...discharge: patient-reported barriers to discharge readiness at an academic medical center. J Hosp Med. 2016;11:610–614. 27079295 10.1002/jhm.2591 PMC12988425

[5] Weiss ME Yakusheva O Bobay KL. Quality and cost analysis of nurse staffing, discharge preparation, and postdischarge utilization. Health Serv Res. 2011;46:1473–1494. 21517836 10.1111/j.1475-6773.2011.01267.x PMC3207188

[6] Bobay KL Yakusheva O Weiss ME. Outcomes and cost analysis of the impact of unit-level nurse staffing on post-discharg utilization. Nurs Econ. 2011;29:69–78; 87. 21667673

[7] Centers for Medicare & Medicaid Services. Fact Sheet - FY 2023 Hospital Inpatient Prospective Payment System (IPPS) and Long-Term Care Hospital Prospective Payment System (LTCH PPS) Final Rule — CMS-1771-F. Centers for Medicare and Medicaid; 2023 https://www.cms.gov/newsroom/fact-sheets/fy-2023-hospital-inpatient-prospective-payment-system-ipps-and-long-term-care-hospitals-ltch-pps

[8] Guo JW Wallace AS Luther BL. Psychometric evaluation of the screener for intensifying community referrals for health. Eval Health Prof. 2022;45:270–276. 34235988 10.1177/01632787211029360 PMC8741833

[9] Wallace AS Luther B Guo JW. Implementing a social determinants screening and referral infrastructure during routine emergency department visits, Utah, 2017-2018. Prev Chronic Dis. 2020;17:E45. doi:10.5888/pcd17.190339 32553071 PMC7316417

[10] Wallace AS Luther BL Sisler SM. Integrating social determinants of health screening and referral during routine emergency department care: evaluation of reach and implementation challenges. Implement Sci Commun. 2021;2:114. doi:10.1186/s43058-021-00212-y 34620248 PMC8499465

[11] Wallace AS Pierce NL Davisson E. Social resource assessment: application of a novel communication tool during hospital discharge. Patient Educ Couns. 2019;102:542–549. 30287147 10.1016/j.pec.2018.09.022

[12] Weiss M Yakusheva O Bobay K. Nurse and patient perceptions of discharge readiness in relation to postdischarge utilization. Med Care. 2010;48:482–486. 20393364 10.1097/MLR.0b013e3181d5feae

[13] Weiss ME Costa LL Yakusheva O. Validation of patient and nurse short forms of the Readiness for Hospital Discharge Scale and their relationship to return to the hospital. Health Serv Res. 2014;49:304–317. 23855675 10.1111/1475-6773.12092 PMC3922479

[14] Weiss ME Piacentine LB. Psychometric properties of the Readiness for Hospital Discharge Scale. J Nurs Meas. 2006;14:163–180. 17278337 10.1891/jnm-v14i3a002

[15] Weiss ME Yakusheva O Bobay KL. Effect of implementing discharge readiness assessment in adult medical-surgical units on 30-day return to hospital: The READI Randomized Clinical Trial. JAMA Netw Open. 2019;2:e187387. doi:10.1001/jamanetworkopen.2018.7387 30681712 PMC6484543

[16] Wallace AS Perkhounkova Y Bohr NL. Quality of transition from hospital to home: the influence of nurse- and patient-reported readiness. Clin Nurs Res. 2018;27:129–147. 27635034 10.1177/1054773816669449

[17] Wallace AS Perkhounkova Y Bohr NL. Readiness for hospital discharge, health literacy, and social living status. Clin Nurs Res. 2016;25:494–511. 26787745 10.1177/1054773815624380

[18] Fitzgerald Miller J Piacentine LB Weiss M. Coping difficulties after hospitalization. Clin Nurs Res. 2008;17:278–296. 18927261 10.1177/1054773808325226

[19] Coffey A McCarthy GM. Older people’s perception of their readiness for discharge and postdischarge use of community support and services. Int J Older People Nurs. 2013;8:104–115. 22309350 10.1111/j.1748-3743.2012.00316.x

[20] Damery S Combes G. Evaluating the predictive strength of the LACE index in identifying patients at high risk of hospital readmission following an inpatient episode: a retrospective cohort study. BMJ Open. 2017;7:e016921. doi:10.1136/bmjopen-2017-016921 PMC5726103 28710226

[21] Harris PA Delacqua G Taylor R. The REDCap Mobile Application: a data collection platform for research in regions or situations with internet scarcity. JAMIA Open. 2021;4:ooab078. doi:10.1093/jamiaopen/ooab078 34527889 PMC8435658

[22] Harris PA Taylor R Minor BL. The REDCap consortium: Building an international community of software platform partners. J Biomed Inform. 2019;95:103208. doi:10.1016/j.jbi.2019.103208 31078660 PMC7254481

[23] Harris PA Taylor R Thielke R. Research electronic data capture (REDCap)--a metadata-driven methodology and workflow process for providing translational research informatics support. J Biomed Inform. 2009;42:377–381. 18929686 10.1016/j.jbi.2008.08.010 PMC2700030

[24] Wagner AK Soumerai SB Zhang F. Segmented regression analysis of interrupted time series studies in medication use research. J Clin Pharm Ther. 2002;27:299–309. 12174032 10.1046/j.1365-2710.2002.00430.x

[25] Morgan D. Qualitative content analysis: a guide to paths not taken. Qual Health Res. 1993;3:112–121. 8457790 10.1177/104973239300300107

[26] Lincoln YSGE. Naturalistic Inquiry. Sage; 1985.

[27] Weiner BJ Lewis CC Stanick C. Psychometric assessment of three newly developed implementation outcome measures. Implement Sci. 2017;12:108. doi:10.1186/s13012-017-0635-3 28851459 PMC5576104

[28] Hirschman KB Shaid E McCauley K. Continuity of care: the transitional care model. Online J Issues Nurs. 2015;20:13–27. 26882510

[29] Becker C Zumbrunn S Beck K. Interventions to improve communication at hospital discharge and rates of readmission: a systematic review and meta-analysis. JAMA Netw Open. 2021;4:e2119346. doi:10.1001/jamanetworkopen.2021.19346 34448868 PMC8397933

[30] Jayakody A Bryant J Carey M. Effectiveness of interventions utilising telephone follow up in reducing hospital readmission within 30 days for individuals with chronic disease: a systematic review. BMC Health Serv Res. 2016;16:403. doi:10.1186/s12913-016-1650-9 27538884 PMC4990979

[31] Leppin AL Gionfriddo MR Kessler M. Preventing 30-day hospital readmissions: a systematic review and meta-analysis of randomized trials. JAMA Intern Med. 2014;174:1095–1107. 24820131 10.1001/jamainternmed.2014.1608 PMC4249925

[32] Li R Geng J Liu J. Effectiveness of integrating primary healthcare in aftercare for older patients after discharge from tertiary hospitals-a systematic review and meta-analysis. Age Ageing. 2022;51:afac151. doi:10.1093/ageing/afac151 35753767 PMC9233979

[33] Dhalla IA O’Brien T Morra D. Effect of a postdischarge virtual ward on readmission or death for high-risk patients: a randomized clinical trial. JAMA. 2014;312:1305–1312. 25268437 10.1001/jama.2014.11492

[34] Schnipper JL Samal L Nolido N. The effects of a multifaceted intervention to improve care transitions within an accountable care organization: results of a stepped-wedge cluster-randomized trial. J Hosp Med. 2021;16:15–22. 33357325 10.12788/jhm.3513 PMC7768916

[35] National Academies of Sciences, Engineering, and Medicine; Health and Medicine Division; Board on Health Care Services. Integrating Social Needs Care into the Delivery of Health Care to Improve the Nation’s Health. 2019. https://www.nationalacademies.org/our-work/integrating-social-needs-care-into-the-delivery-of-health-care-to-improve-the-nations-health

