# Supplementary material for: Impact of Social Risk Screening on Discharge Care Processes and Postdischarge Outcomes: A Pragmatic Mixed-Methods Clinical Trial During the COVID-19 Pandemic
Source: Med Care. 2024 Sep 6;62(10):639–49. doi: 10.1097/MLR.0000000000002048 (PMC11373892; doi:10.1097/MLR.0000000000002048)
Supplement: Supplementary file 1 [file mlr-62-639-s001.docx]

Supplemental Digital Content 1

NEEDS Study Physician Interview Guide

INTRODUCTION

Thank you for agreeing to talk with us about your experience with discharging patients. In this interview, when I talk about social needs, I am talking about needs for things such as transportation, food, housing, or household goods. When I talk about social support, I’m talking about patients’ social environment and supports – whether they have someone to help them with tasks common to patients upon hospital discharge such as with transportation, running errands, cooking, bathing, dressing changes, etc.

OPENING QUESTIONS

**Question #2:** What do you see as your role in the assessment and response to patients’ social needs?

1. IF THEY ANSWER THAT THEY HAVE A ROLE: Have you ever encountered any barriers in responding to this information?
2. How important do you feel it is for care teams at the hospital to know about a patient’s social needs and social supports outside of the hospital?
3. What role do you see other members of the care team playing?
   1. Probe if necessary: case managers, doctors, social workers, physical or occupational therapists

**Question #3**. Tell me about how information about patients’ physical and social environment at home was being communicated to you and to the rest of the team in charge of discharge prior to March 15^th^ when we added additional social needs and social support questions to your workflow.

1. Was the information routinely shared among those involved in discharge planning?
   1. If yes: how was the information shared? How often? (if they push back for clarification: a lot, a little, most of the time, etc.) If it was shared, did you feel like this gave the discharge team adequate information? If not, what was missing?
   2. If not, why was the information not shared?

INTERVENTION QUESTIONS

In the NEEDS project, we aimed to assess and communicate information about patients’ physical and social environment known to be important in their ability to successfully transition home. We specifically focused on assessing and communicating patients’ social needs (for things like transportation, food, housing) and social support (whether patients have someone at home to help them with common self-management tasks). As of March 15^th^, a social needs and personal resource assessment, completed by case managers, has been made visible in Epic via the Discharge Planning tab (also known as the IP Discharge Readiness Assessment). When case management determines the patient will not have sufficient social support after discharge we communicated that result to Attendings through In Basket messaging in Epic.

**Question #3**: Since March, have you viewed the social needs and supportive resources information displayed in Epic?

If YES -

**3a**. Was the information useful in helping plan patient discharge? Can you share some specific examples of how you and your team used the information?

**3a.1**. (If they didn’t already mention anything along these lines) How did it impact discharge timing and destination?

**3b**. Did you communicate the information to others on the team?

**3b.1**. If yes, can you tell me more about that? With whom did you discuss the information and why?

**3c**. Did you discuss the information with patients and/or their families?

**3c.1**. If yes, can you tell me more about that experience? What would facilitate communication among team members? Who do you think should see the social needs information when a patient is admitted?

**3d.** Is there more you would like to know about the patient to make the social needs information useful to you? What do you think would be useful?

**3e**. What other recommendations do you have that would help to integrate social needs information into discharge planning?

[Go to question #4]

If NO -

**3f**. Do you know about the social needs and supportive resources information displayed in Epic?

**3f.1 I**f yes, is there a reason you haven’t viewed or used the information? What would inspire you to look at information about a patient’s social needs and resources?

3f.2 If no, would you be interested in using information like this in your discharge planning? How would you anticipate using the information?

[Go to question #5]

**Question #4:** Before the Epic social needs and resource information, how did you come to know details about patients’ physical and social environment at home that may impact discharge planning?

4a. Did you know this on every patient? If not, on which patients did you know this information?

4b. Did you feel like this gave you adequate information? If not, what was missing?

4c. Were patients’ social needs and supportive resources shared among those involved in discharge planning? With whom, when, and why? Were there gaps in information sharing? If so, can you tell me more about that?

**Question #5:** How do you typically come about knowing social information about patients?

5a. Do you know this on every patient? If not, on which patients do you know this information?

5b. Is there additional social information you want to know?

5c. How are patients’ social needs and supportive resources shared among those involved in discharge planning? With whom? When?

5d. Is there something you think could be done to make the new Epic social needs and resources information useful to you? What might facilitate communication among discharge team members? Who do you think should see the social needs information?

PANDEMIC QUESTIONS

**Question #6:** Overall, knowing what your workload was like before the pandemic, how attainable would this have been?

**6.a.** How has the pandemic impacted your interactions with patients?

**6.b.** Has the pandemic changed your view of social needs and the incorporation of social resource information assessments into hospital systems? If so, how?

WRAP-UP QUESTIONS

**Question #6:** Many institutions are working to integrate social determinants of health questions – like the NEEDS social needs and social support questions – into clinical care. How do you feel about this?

**Question #7**: Is there anything else you would like to share with us about whether and how patients’ social information is incorporated into discharge planning processes?
