# Supplementary material for: Impact of Social Risk Screening on Discharge Care Processes and Postdischarge Outcomes: A Pragmatic Mixed-Methods Clinical Trial During the COVID-19 Pandemic
Source: Med Care. 2024 Sep 6;62(10):639–49. doi: 10.1097/MLR.0000000000002048 (PMC11373892; doi:10.1097/MLR.0000000000002048)
Supplement: Supplementary file 2 [file mlr-62-639-s002.docx]

**Supplemental Digital Content 2.**Physician Interview Participants (N=15)

| **Physician Participants** | n (%) |
| --- | --- |
| Age |  |
| 18-30 | 2 (13.3) |
| 31-45 | 10 (66.7) |
| 46-60 | 3 (20) |
| Gender |  |
| Female | 8 (53.3) |
| Male | 7 (46.7) |
| Race |  |
| Asian | 3 (20) |
| White | 11 (73.3) |
| Multiracial (Asian and White) | 1 (6.7) |
| Ethnicity |  |
| Non-Hispanic | 14 (93.3) |
| Hispanic | 1 (6.7) |
| Specialty |  |
| General Surgery | 8 (53.3) |
| Internal Medicine | 7 (46.7) |
